# Clinical presentation and initial management of Black men and White men with prostate cancer in the United Kingdom: the PROCESS cohort study

**DOI:** 10.1038/sj.bjc.6605461

**Published:** 2009-11-24

**Authors:** S Evans, C Metcalfe, B Patel, F Ibrahim, K Anson, F Chinegwundoh, C Corbishley, D Gillatt, R Kirby, G Muir, V Nargund, R Popert, P Wilson, R Persad, Y Ben-Shlomo

**Affiliations:** 1 Department of Social Medicine, University of Bristol, 39 Whatley Road, Bristol, BS8 2PS, UK; 2 Department of Urology, University Hospitals Bristol NHS Foundation Trust, Bristol Royal Family, Upper Maudlin Street, Bristol, BS2 8HW, UK; 3 Department of Urology and Pathology, St George's Hospital, Blackshaw Road, Tooting, London, SW17 0QT, UK; 4 Department of Urology, Barts and the London NHS Trust, St Bartholomew's Hospital, West Smithfield, London, EC1A 7BE, UK; 5 Department of Urology, Newham University Hospital NHS Trust, Glen Road, London, E13 8SL, UK; 6 Department of Urology, North Bristol NHS Trust, Southmead Hospital, Southmead Road, Bristol, BS10 5NB, UK; 7 Department of Urology, King's College Hospital NHS Trust, Denmark Hill, London, SE5 9RS, UK; 8 Department of Urology, Guy's and St Thomas’ NHS Foundation Trust, Guy's Hospital, Great Maze Pond, London, SE1 9RT, UK; 9 Bristol Oncology Centre, Bristol Haematology and Oncology Centre, Horfield Road Bristol, Bristol, BS2 8ED, UK

**Keywords:** clinical management, diagnosis, epidemiology, ethnicity, prostate cancer

## Abstract

**Background:**

In the United States, Black men have a higher risk of prostate cancer and worse survival than do White men, but it is unclear whether this is because of differences in diagnosis and management. We re-examined these differences in the United Kingdom, where health care is free and unlikely to vary by socioeconomic status.

**Methods:**

This study is a population-based retrospective cohort study of men diagnosed with prostate cancer with data on ethnicity, prognostic factors, and clinical care. A Delphi panel considered the appropriateness of investigations and treatments received.

**Results:**

At diagnosis, Black men had similar clinical stage and Gleason scores but higher age-adjusted prostate-specific antigen levels (geometric mean ratio 1.41, 95% confidence interval (95% CI): 1.15–1.73). Black men underwent more investigations and were more likely to undergo radical treatment, although this was largely explained by their younger age. Even after age adjustment, Black men were more likely to undergo a bone scan (odds ratio 1.37, 95% CI: 1.05–1.80). The Delphi analysis did not suggest differential management by ethnicity.

**Conclusions:**

This UK-based study comparing Black men with White men found no evidence of differences in disease characteristics at the time of prostate cancer diagnosis, nor of under-investigation or under-treatment in Black men.

A recent systematic review comparing Black men with White men diagnosed with prostate cancer found evidence of poorer prognosis in Black men (Evans *et al*, 2008). All studies included in the meta-analysis were based in the United States where, on average, Black men occupy less privileged socioeconomic positions (Shapiro and Oliver, 1996; Jones *et al*, 2008) and where access to health services is largely determined by the patient's ability to pay. Hence, the poorer prognosis in Black men is not necessarily due to a more aggressive disease type. Alternative possibilities are that Black men in the United States are diagnosed later because of poorer access to diagnostic services (Institute of Medicine, 2001), may have a poverty-related higher risk of comorbidity and hence less resilience to disease progression (Davey Smith *et al*, 1998), and may have their disease managed less aggressively (Shavers and Brown, 2002; Shavers *et al*, 2004a, 2004b). Meta-analysis of findings from studies accounting for clinical characteristics at diagnosis and, in some cases, socioeconomic status suggested a reduced but still apparent disadvantage for Black men with prostate cancer in terms of prostate-cancer-specific mortality and biochemical recurrence (Evans *et al*, 2008). In fact, few studies measured socioeconomic status, and all relied on ecological measures as a proxy indicator of individual status.

The possibility remains that a better control of confounding factors may completely account for the observed poorer prognosis in US-resident Black men with prostate cancer. Alternatively, the examination of cohorts with minimal ethnic variation in socioeconomic factors and clinical management could result in greater confidence that any differences in prognosis are more likely due to biological factors. The Prostate Conditions in Ethnic Subgroups (PROCESS) cohort is based in Southern England, where free medical care is available to all from the UK National Health Service. Studies of this cohort have shown a higher rate of incident prostate cancer in Black men compared with White men (Ben-Shlomo *et al*, 2008), and although Black men were more likely to work in manual occupations (81 *vs* 67%) and to live in less-affluent areas, there were no marked differences in knowledge of prostate cancer, in delays before seeking medical attention, in the average prostate-specific antigen (PSA) level at diagnosis, or in levels of comorbidity (Metcalfe *et al*, 2008).

In this analysis, we compare diagnostic investigations, the clinical stage of disease at presentation, and initial management between Black men and White men diagnosed with prostate cancer. Variations in management may be difficult to interpret, as one group may be either managed too aggressively or the other group may be under-managed. Therefore, we used a ‘Delphi’ consensus approach to allow us to classify whether management was appropriate for a large number of clinical scenarios (Shekelle *et al*, 1998) and then compared this with our observed patterns of care by ethnicity. This approach allows one to conclude whether any observed variations in management between Black men and White men are accounted for by differences in clinical need between the two groups of men.

## Participants and methods

The Prostate Conditions in Ethnic Subgroups, a population-based retrospective cohort study, has been previously described (Ben-Shlomo *et al*, 2008; Metcalfe *et al*, 2008). Cases of prostate adenocarcinoma were identified from among males residing in four study areas (namely North Bristol, South-West London, South-East London, and North-East London) during 1995–1999 (Bristol) or during 1997–2001 (London). Possible cases of prostate cancer were identified from the following sequence of sources: (1) pathology databases and (North-West London only) a urology department database, (2) hospital discharge diagnosis files, (3) PSA records >10 ng ml^−1^, and (4) Cancer Registry (Bristol only). In cases in which histological proof of cancer was not available (e.g., having relied on PSA records), a panel of at least four urologists classified a case vignette as a ‘clinical’ (non-histological proven) case of prostate cancer, or excluded it because of lack of evidence.

The South-West Multi-Centre Research Ethics Committee approved the PROCESS study.

Men known to be alive at the time of the study were asked to complete a questionnaire including the 2001 census questions on ethnicity, with the next-of-kin being contacted if the man had died more than 6 months ago. Questionnaire information determined ethnicity for 37% of Black men and 45% of White men. If a man's ethnicity remained undetermined, we referred in turn to hospital records (62% of Black men, 50% of White men) and place of birth recorded on the death certificate (1% of Black men, 5% White men).

Trained research nurses reviewed hospital records using a standard proforma, extracting information on PSA measurements, histological investigations, investigations aimed at determining cancer stage, and initial management strategies. The North-East London centre was restricted by time and reviewed all case notes for Black men, and a random sample of 50% of case notes for White men. Taking this into account, three centres completed reviews for more than 85% of cases, whereas the North-East London centre completed reviews for 76%.

An ecological measure of socioeconomic position was obtained by linking each man's home postcode to the corresponding 1998 electoral ward (http://www.edina.ac.uk/), then to the Index of Multiple Deprivation (IMD) score for the year 2000 (http://www.neighbourhood.statistics.gov.uk/). Six domains (namely income; employment; health deprivation and disability; education, skills, and training; housing; and geographical access to services) determine the index score for an area, with higher scores indicating greater deprivation. Occupation, available for 1249 of the 1461 (85%) men whose medical records were reviewed, formed the basis of an individual measure of socioeconomic position, with occupation being classified as manual or non-manual according to the Registrar General's scheme (see http://www.statistics.gov.uk/).

Measures of PSA level and tumour differentiation from the time of diagnosis and before commencing any treatment were identified. The following categories of tumour differentiation were distinguished: (1) Gleason scores (Gilliland *et al*, 2001) up to 6, well differentiated, or moderately differentiated; (2) a Gleason score of 7; and (3) a Gleason score of 8 or more, or poorly differentiated. The use of MRI or CT scans to identify metastases was noted, with stage at diagnosis being recorded according to the TNM system (Ohori *et al*, 1994). We distinguished three categories: (1) tumour confined to the prostate, T1/T2; (2) tumour spread to structures adjacent to the prostate, T3/T4/N1; and (3) tumour spread to distant structures, M1. Finally, for this study, the initial management strategy was determined with the following four categories distinguished: (1) radical prostatectomy with curative intent; (2) radiotherapy with curative intent; (3) hormone therapy, including orchidectomy, with the intention of slowing progression; and (4) conservative treatment, including watchful waiting, palliative treatment, and no treatment.

A Delphi sub-study investigated the appropriate use of diagnostic investigations and management strategies. Four urologists and one oncologist working in the PROCESS study centres were asked to independently consider 126 hypothetical patients, constructed as combinations of age (<65, 65–74, and 75+ years), PSA (<20, 20–99, 100+ ng ml^−1^), disease stage (localised, locally advanced, and metastatic disease), Gleason score (<5, 5–7, and 8+), and comorbidity (low and moderate/high). Each hypothetical patient was rated as being appropriate for a bone scan, a CT scan, radical prostatectomy, radical radiotherapy, hormones only, and watchful waiting on a 1–9 scale, with 1–3 being inappropriate, 4–6 being equivocal, and 7–9 being appropriate. For each hypothetical patient, a median of the five ratings was taken for the appropriateness of each procedure. Each PROCESS cohort member was then matched to a hypothetical patient according to the above-mentioned categories of age, PSA, stage, grade, and comorbidity, and was thus matched to median appropriateness ratings for different investigations and approaches. In situations in which a match with a hypothetical patient was not available, the PROCESS cohort member was not included in this part of the analysis.

### Statistical analysis

Owing to their positively skewed distribution, PSA levels are presented as medians with 90% ranges, and the association with ethnicity is presented as a ratio of geometric means. Geometric means are similar in value to medians, and their ratio can be estimated using standard regression methods applied to natural log-transformed PSA measurements.

Multivariable regression models estimated the associations between race and binary factors (logistic regression), and ordered categorical factors (ordered logistic regression) (Kirkwood and Sterne, 2003). Odds ratios were centre and age adjusted by including each of these covariates in regression models as four dummy variables, distinguishing the five study centres and five age categories. A further adjustment for socioeconomic factors was attempted by adding three dummy variables to multivariable models, distinguishing quartiles of IMD scores, and a single dummy variable distinguishing men in manual occupations from those in non-manual occupations.

Further analyses compared the use of each diagnostic investigation and treatment strategy between Black men and White men, each analysis being stratified by how appropriate the procedure was judged to be for a man by the Delphi panel. For each procedure, an interaction test addressed the null hypothesis of equal ethnic differences across categories of appropriateness. The interaction test was implemented by adding extra covariates to the regression model, which would capture any variation in the ethnic difference across categories of appropriateness, and then testing the null hypothesis that the coefficients for those extra terms were all zero on the log scale. For these analyses, crude- and age-adjusted analyses are presented, the reduced sample size not being sufficient for centre adjustment within strata. For the same reason, age is included as a linear continuous covariate in these analyses.

Agreement between Delphi panel members with regard to the use of three broad categories (inappropriate, equivocal, and appropriate) for each investigation and procedure was estimated using an unweighted, multi-rater *κ*-statistic applied to data from all 126 hypothetical patients (Landis and Koch, 1977).

CI denotes confidence interval, and all *P*-values are two-tailed. Analyses were undertaken using Stata version 10 (StataCorp, College Station, TX, USA, 2007).

## Results

As previously reported, Black men presented at a younger age (mean 67.9 years, s.d. 7.3 years, *n*=547) compared with White men (mean 73.3 years, s.d. 8.8 years, *n*=1319), with the observed distributions being consistent with normal distributions in the population in each case (Metcalfe *et al*, 2008). Review of hospital records was completed and provided data for 473 (86%) Black men and 988 (91% of the 1098 reviews planned) White men. Where available, the median pre-treatment PSA level was 25 ng ml^−1^ for Black men (90% range 4.8–822 ng ml^−1^; *n*=436) and 23 ng ml^−1^ for White men (90% range 3.8–1325 ng ml^−1^; *n*=863). The centre-adjusted analysis of PSA levels provided no convincing evidence of a difference (geometric mean ratio 1.11, 95% CI: 0.91–1.35, *P*=0.32), but there was strong evidence of higher levels in Black men than in White men of similar age (geometric mean ratio 1.41, 95% CI: 1.15–1.73, *P*=0.001). Only 16% of Black men and 11% of White men were diagnosed in the absence of symptoms, after a PSA test.

A histological report was available from the time of diagnosis for more than 90% of men, with the distribution of reported Gleason scores being comparable between Black men and White men (Table 1). There was some evidence that Black men were more likely to undergo a bone scan or a CT scan, but for the latter, the age-adjusted analysis suggested that this was because of Black men being diagnosed at a younger age (Table 1). The distribution of cancer stages at diagnosis was very similar between Black men and White men, with three-quarters of both groups being diagnosed while their disease was still localised (Table 1). An additional adjustment of these analyses for the occupation- and area of residence-based measures of socioeconomic position did not affect the estimated differences between Black men and White men (data not shown).

Of those men whose disease stage was recorded, the initial treatment plan was determined by reviewing the medical records of 415 out of 437 (95%) Black men, and 851 out of 891 (96%) White men. The majority of men diagnosed with cancer spread to adjacent structures (T3/T4/N1) were initially treated with hormones (20 out of 28 (71%) Black men and 34 out of 54 (63%) White men) or radiotherapy (8 out of 28 (29%) Black men and 10 out of 54 (19%) White men). Men diagnosed with metastatic cancer were predominantly treated with hormones (68 out of 73 (93%) Black men and 152 out of 164 (93%) White men), with a handful receiving palliative treatment (4 out of 73 (5%) Black men and 9 out of 164 (5%) White men). All four noted management strategies were used in the case of men diagnosed with localised cancer (Table 2). There was evidence that Black men were more likely than White men to undergo a curative treatment strategy for their localised cancer, but the age-adjusted analyses indicated that this was because of the younger age of Black men at diagnosis (Table 2). In fact, although the evidence was very weak and consistent with chance, Black men seemed to be less likely to undergo curative treatment when compared with their same-age White counterparts. Additional adjustment for occupation- and area of residence-based measures of socioeconomic position reduced the observed difference between Black men and White men in curative treatment (odds ratio 0.81, 95% CI: 0.54–1.24), although this analysis was only possible for men with a known occupation (720 out of 937 (77%)).

Supplementary Appendix 1 presents the hypothetical patients from the Delphi exercise who most commonly matched to PROCESS cohort members, with the appropriateness ratings for investigations and management strategies. There was fair-to-moderate agreement, measured using the *κ*-statistic, between Delphi panel members on the appropriateness of interventions for the set of hypothetical patients (prostatectomy 0.31, radiotherapy 0.44, watchful waiting 0.18). Agreement among panel members with regard to investigations was at chance levels, although for bone scanning, this was because of all five panel members considering a bone scan to be appropriate for the large majority of patients, with this lack of variation not allowing inter-rater agreement to be apparent.

In Table 3, those cohort members matched to a hypothetical patient are stratified by their suitability for each investigation and management strategy. The odds of undergoing each procedure are then compared between Black men and White men within each stratum of suitability. There is no convincing evidence that the differences between Black men and White men observed in Table 2 varied by the judged appropriateness of the procedures (all interaction *P*-values are >0.3). With regard to crude comparisons, there was a trend for the higher rate of radiotherapy in Black men to be particularly apparent among men for whom the treatment was judged appropriate. With regard to age-adjusted analyses, the reduced rates of radiotherapy and surgery in Black men when compared with same-age White men (Table 2) seemed to occur in clinical scenarios in which the decision to undertake these procedures was equivocal.

The Delphi committee recommended bone scanning for all men. This is inconsistent with recent UK National Institute for Health and Clinical Excellence (NICE) guidelines, which recommend against routine bone scanning for men with ‘low risk’ (PSA <0 ng ml^−1^ and Gleason score of ⩽6) localised prostate cancer (National Institute for Health Clinical Excellence, 2008). Owing to this reason, as well as to assist the interpretation of our findings, we stratified the comparison of bone scan and CT scan rates in men with clinically localised disease by the NICE risk categories. Low risk is as defined above, intermediate risk is a PSA level between 10 and 19.99 or a Gleason score of 7, and high risk is a PSA level >20 or a Gleason score of ⩾8. When compared with White men of the same age, Black men were more likely to undergo a bone scan in all three risk categories (Table 4), and a CT scan in the intermediate- and high-risk categories, but the wide CIs indicate that these differences could have occurred by chance. There was no evidence that the association between ethnicity and undergoing either investigation varied by the NICE risk category (*P*-value for interaction, 0.42 and 0.27 for bone scanning and CT scanning, respectively).

## Discussion

The UK-based PROCESS cohort provides no evidence of Black men being diagnosed with more advanced prostate cancer than White men, although they had greater age-adjusted PSA levels. Black men were more likely to undergo CT and MRI scans, and were more likely to undergo curative treatment for localised disease, but this was entirely accounted for by their younger age at diagnosis. There is no evidence that Black men were managed less appropriately than their same-age White counterparts, but in this Delphi sub-study, the CIs around estimates are relatively wide. There was evidence that White men were less likely to undergo a bone scan, although this may be because of the older average age of White men, allowing residual confounding by comorbidity.

Surveillance, Epidemiology, and End Results (SEER) data on tumour grade at diagnosis are available from the California Cancer Registry for the period between 1995 and 2004, in which they found 24.3% of Black men and 22.9% of White men to have poorly differentiated or undifferentiated tumours (Robbins *et al*, 2007). Hence, around the turn of the century, similar proportions of Black men and White men were diagnosed with aggressive tumours in the United States, and in fact, these figures are very similar to the proportions of men we found in the United Kingdom with equivalent Gleason scores of between 8 and 10 at diagnosis (Table 1). Compared with our data (Table 1), a greater proportion of Californian men were diagnosed with localised (T1/T2) cancer, with 79.2% of Black men and 84.3% of White men diagnosed while their tumour was localised (Robbins *et al*, 2007). A smaller proportion of Californian men were diagnosed with metastasised disease (6.4% of Black men and 4.0% of White men) (Robbins *et al*, 2007). These differences are potentially because of the greater use of PSA testing in the United States during this period.

In contrast to diagnostic procedures, more differences were apparent in initial treatment. About a third of Californian men underwent surgery, with slightly more White men (35.7%) than Black men (32.6%) being managed initially in this manner (Robbins *et al*, 2007). Similarly, radiotherapy was the initial treatment for 32.7% of White men and 30.0% of Black men (Robbins *et al*, 2007). The greater use of these potentially curative treatments in the United States is, in part, because of the younger average age at diagnosis (median age at diagnosis in US White men is 69 years and that in US Black men is 66 years (Karami *et al*, 2007)), and perhaps, in part, because of a more rapid uptake of prostatectomy and radiotherapy in the United States during the period studied, when they were relatively new interventions (Collin *et al*, 2008). In the United Kingdom, although a curative treatment plan was less common (Table 2), the decision to follow such a plan was taken more equitably between Black men and White men, with no strong evidence of a difference once the age difference was taken into account. In fact, there is weak evidence in our data of Black men being more likely than White men to undergo those investigations and curative treatments if considered to be clinically appropriate.

The PROCESS study has collected detailed demographic and clinical information from medical records and questionnaires on a cohort of men diagnosed with prostate cancer. A Delphi sub-study has provided information on the medical decision-making process of clinicians working at hospitals in which the cohort men were diagnosed and treated. The resulting data are considerably richer than those available to cancer registries, in spite of the PROCESS cohort being smaller; therefore, the estimates from our stratified analyses will be less precise. Measurement error may be a concern for data extracted from routine medical records, although ethnic variation in record keeping practice is unlikely. Finally, PROCESS is a retrospective cohort study; hence, the men were diagnosed and treated some time before the Delphi panel considered it to be appropriate practice. However, although the Delphi panel recommendations may vary over time, the variation will not be associated with race, as the vignettes do not contain that information.

To conclude, in this UK-based cohort, Black men and White men are diagnosed with prostate cancer at comparable points in the natural history of the disease, as determined by tumour grade and tumour stage, although with higher age-adjusted PSA levels. Once younger age at diagnosis is taken into account, Black men are as likely to undergo CT or MRI scanning as White men, and are as likely to have localised disease treated with curative intent. White men were noted to be less likely to receive a bone scan. This comparability between the experiences of Black men and White men, coupled with detailed information on socioeconomic position available for the PROCESS cohort, will assist interpretation when comparing survival between Black men and White men after a diagnosis of prostate cancer.

## Figures and Tables

**Table 1:** Investigations and disease characteristics at diagnosis

	**Black men**	**White men**	**Centre adjusted**		**Centre and age adjusted**	
	***n* (%)**	***n* (%)**	**OR (95% CI)**	***P*-value**	**OR (95% CI)**	***P*-value**
Has hospital records review (*n*=1461)	473	988				
						
*Histological diagnosis (n=1437)*						
No	15 (3)	58 (6)				
Yes	451 (97)	913 (94)	1.27 (0.68, 2.36)	0.45	0.81 (0.41, 1.58)	0.53
						
*Gleason score at diagnosis (n=1309)*						
⩽6	242 (55)	486 (56)				
7	105 (24)	190 (22)				
8/10	91 (21)	195 (22)				
Trend per category increase			0.95 (0.75, 1.19)	0.66	1.08 (0.85, 1.37)	0.54
						
*Bone scan (n=1461)*						
Not recorded^a^	45 (10)	91 ( 9)				
No^a^	77 (16)	268 (27)				
Yes	351 (74)	629 (64)	1.50 (1.16, 1.95)	0.002	1.37 (1.05, 1.80)	0.02
						
*CT investigation (n=1461)*						
Not recorded^a^	118 (25)	209 (21)				
No^a^	268 (57)	639 (65)				
Yes	87 (18)	140 (14)	1.33 (0.97, 1.83)	0.079	1.03 (0.74, 1.45)	0.85
						
*Stage at diagnosis (n=1328)*						
T1/T2	332 (76)	663 (74)				
T3/T4/N1	30 (7)	56 (6)				
M1	75 (17)	172 (19)				
Trend per category increase			1.02 (0.77, 1.35)	0.91	1.13 (0.84, 1.51)	0.43

Abbreviations: CI, confidence interval; CT=computed tomography; OR=odds ratio.

a These two groups combined and used as the comparison in calculating the OR.

**Table 2:** Initial treatment of men diagnosed with T1/T2 cancer

			**Adjusted for**					
	**Black men**	**White men**	**Centre (*n*=947)**		**Centre and age (*n*=947)**		**Centre, age, grade, stage, and comorbidity (*n*=846)**	
	***n* (%)**	***n* (%)**	**OR (95% CI)**	***P*-value**	**OR (95% CI)**	***P*-value**	**OR (95% CI)**	***P*-value**
Has T1/T2 cancer	332	663						
and initial treatment recorded	314 (95)	633 (95)						
No treatment	50 (16)	188 (30)						
Hormone therapy	118 (38)	249 (39)						
Curative radiotherapy	104 (33)	123 (19)	1.67 (1.20, 2.32)	0.002	0.85 (0.58, 1.23)	0.39	0.77 (0.51, 1.14)	0.19
Curative surgery	42 (13)	73 (12)	1.44 (0.92, 2.27)	0.114	0.67 (0.38, 1.16)	0.15	0.79 (0.42, 1.48)	0.45
								
Curative radiotherapy or surgery	146 (46)	196 (31)	1.57 (1.17, 2.10)	0.003	0.74 (0.52, 1.05)	0.088	0.71 (0.49, 1.03)	0.074

Abbreviations: CI, confidence interval; OR=odds ratio.

Comparisons are against the combination of no treatment+hormone therapy. Dummy covariates distinguish the five centres, three age groups (44–68, 69–76, and 77–94 years), three groups of Gleason scores (⩽6, 7, and 8+), two stages (T1 and T2), and three groups of Charlson scores (0, 1, and 2+).

**Table 3:** Black men and White men undergoing different procedures, by Delphi judgement on the appropriateness of those procedures

			**Underwent procedure; Black men/White men**	
	**Underwent procedure *N* (%)**		**Crude estimates**	**Age adjusted**
**Delphi opinion on appropriateness of procedure for patient:**	**Black men**	**White men**	**OR (95% CI)**	**OR (95% CI)**
*CT scan (n=803)*				
Inappropriate	27/108 (25)	46/234 (20)	1.36 (0.79, 2.34)	1.05 (0.60, 1.87)
Equivocal	23/105 (22)	39/229 (17)	1.37 (0.77, 2.43)	1.10 (0.60, 2.02)
Appropriate	14/55 (25)	12/72 (17)	1.71 (0.72, 4.06)	1.64 (0.69, 3.94)
*Interaction P-value*			0.90	0.73
				
*Surgical treatment (n=711)* a				
Inappropriate	0/112 (0)	6/323 (2)		
Equivocal	7/48 (15)	11/ 84 (13)	1.13 (0.41, 3.15)	0.43 (0.12, 1.57)
Appropriate	31/58 (53)	40/86 (47)	1.32 (0.68, 2.57)	1.36 (0.62, 2.97)
*Interaction P-value*			0.81	0.15
				
*Radiological treatment (n=828)* b				
Inappropriate	1/48 (2)	2/100 (2)	1.04 (0.09, 11.79)	0.58 (0.05, 7.12)
Equivocal	14/54 (26)	24/134 (18)	1.60 (0.76, 3.40)	0.74 (0.31, 1.78)
Appropriate	82/175 (47)	89/317 (28)	2.26 (1.54, 3.32)	1.27 (0.83, 1.96)
*Interaction P-value*			0.62	0.63
				
*Hormone treatment (n=923)*				
Inappropriate	5/58 (9)	4/ 74 (5)	1.65 (0.42, 6.45)	1.86 (0.46, 7.59)
Equivocal	35/127 (28)	77/260 (30)	0.90 (0.56, 1.45)	1.17 (0.71, 1.94)
Appropriate	96/130 (74)	196/274 (72)	1.12 (0.70, 1.80)	1.44 (0.86, 2.40)
*Interaction P-value*			0.64	0.56
				
*No active treatment (n=923)*				
Inappropriate	6/142 (4)	42/289 (15)	0.26 (0.11, 0.63)	0.40 (0.16, 1.01)
Equivocal	23/117 (20)	76/209 (36)	0.43 (0.25, 0.73)	0.65 (0.36, 1.16)
Appropriate	15/56 (27)	41/110 (37)	0.62 (0.30, 1.25)	0.84 (0.39, 1.79)
*Interaction P-value*			0.32	0.62

Abbreviations: CI, confidence interval; CT=computed tomography; OR=odds ratio.

a Men undergoing radiotherapy are excluded from the denominators.

b Men undergoing surgery are excluded from the denominators.

**Table 4:** Black men and White men undergoing diagnostic procedures, by the NICE guidance risk categories for men with clinically localised disease, and clinically advanced disease (T3/T4)

			**Underwent investigation; Black/White men**	
	**Underwent procedure *N* (%)**		**Crude estimates**	**Age adjusted**
***NICE risk category* a**	**Black men**	**White men**	**OR (95% CI)**	**OR (95% CI)**
*Bone scanning (n=1068)*				
Low risk	39/64 (61)	73/133 (55)	1.28 (0.70, 2.35)	1.10 (0.59, 2.07)
Intermediate risk	58/72 (81)	99/147 (67)	2.01 (1.02, 3.59)	1.85 (0.92, 3.73)
High risk	171/183 (93)	277/337 (82)	3.09 (1.61, 5.90)	1.84 (0.91, 3.69)
Clinically advanced	34/39 (87)	71/93 (76)	2.11 (0.73, 6.04)	1.50 (0.50, 4.53)
*Interaction P-value*			0.29	0.42
				
*CT scanning* (*n=796)*				
Low risk	9/56 (16)	25/121 (21)	0.74 (0.32, 1.70)	0.58 (0.24, 1.39)
Intermediate risk	20/66 (30)	22/123 (18)	2.00 (1.00, 4.01)	1.94 (0.94, 4.02)
High risk	43/144 (30)	57/286 (20)	1.71 (1.08, 2.71)	1.24 (0.76, 2.02)
Clinically advanced	7/35 (20)	17/88 (19)	1.04 (0.39, 2.79)	0.98 (0.36, 2.69)
*Interaction P-value*			0.24	0.27

Abbreviations: CI=confidence interval; CT=computed tomography; NICE=National Institute for Health and Clinical Excellence; OR=odds ratio.

a Low risk: PSA <10 ng ml^−1^ and Gleason score of ⩽6; intermediate risk: PSA between 10 and 19.99 or Gleason score of 7; high risk: PSA >20 or Gleason score of ⩾8.
