# Supplementary material for: Clinical presentation and initial management of Black men and White men with prostate cancer in the United Kingdom: the PROCESS cohort study
Source: Br J Cancer. 2009 Nov 24;102(2):249–54. doi: 10.1038/sj.bjc.6605461 (PMC2816646; doi:10.1038/sj.bjc.6605461)
Supplement: Supplementary Information [file 6605461x1.doc]

**ONLINE ONLY: The PROCESS Study Group**

The PROCESS study group included; Bristol (P Abrams, D Dickerson, S Falk, R Feneley, D Gillatt, C Gingell, J Graham, F Keeley, H Newman, R Persad, J Probert, H Schwaibold, G Sibley, A Timoney, R Wells, T Whittlestone, M Wright), SW London (C Anderson, K Anson, M Bailey, R Kirby) SE London (D Cahill, P Dasgupta, J Glass, S Khan, G Muir, T O'Brien, R Popert, J Poulsen, P Thompson, R. Tiptaft, K Walsh), NE London (N Buchholz, F Chinegwundoh, C Fowler, I Junaid, V Nargund, A Paris, J Pati,), Sheffield University (D Dorling B Thomas).

**ONLINE ONLY: Appendix 1.** Common scenarios (all matching >20 PROCESS men) with their Delphi recommendations. A bone scan was considered appropriate for all these men.

| **Number of men fitting the scenario** | **Age (years)** | **PSA (ng/ml)** | **Gleason score** | **Stage** | **Co-morbidity** | **CT scan** | **Surgery** | **Radiotherapy** | **Hormones** | **Conservative treatment** |
| --- | --- | --- | --- | --- | --- | --- | --- | --- | --- | --- |
| 27 | <65 | <20 | <5 | Localized | Low | Inappropriate | Appropriate | Appropriate | Equivocal | Appropriate |
|  |  |  |  |  |  |  |  |  |  |  |
| 60 | <65 | <20 | 5-7 | Localized | Low | Inappropriate | Appropriate | Appropriate | Inappropriate | Equivocal |
| 28 | <65 | <20 | 5-7 | Localized | Mod/high | Appropriate | Equivocal | Appropriate | Inappropriate | Equivocal |
|  |  |  |  |  |  |  |  |  |  |  |
| 24 | 65-74 | <20 | <5 | Localized | Low | Equivocal | Appropriate | Appropriate | Inappropriate | Equivocal |
| 21 | 65-74 | <20 | <5 | Localized | Mod/high | Equivocal | Appropriate | Equivocal | Inappropriate | Appropriate |
|  |  |  |  |  |  |  |  |  |  |  |
| 78 | 65-74 | <20 | 5-7 | Localized | Low | Equivocal | Appropriate | Appropriate | Equivocal | Appropriate |
| 47 | 65-74 | <20 | 5-7 | Localized | Mod/high | Appropriate | Equivocal | Appropriate | Equivocal | Equivocal |
|  |  |  |  |  |  |  |  |  |  |  |
| 39 | 65-74 | 20-99 | 5-7 | Localized | Low | Equivocal | Equivocal | Appropriate | Appropriate | Equivocal |
| 37 | 65-74 | 20-99 | 5-7 | Localized | Mod/high | Inappropriate | Inappropriate | Equivocal | Equivocal | Inappropriate |
|  |  |  |  |  |  |  |  |  |  |  |
| 49 | 75+ | <20 | 5-7 | Localized | Low | Inappropriate | Equivocal | Appropriate | Equivocal | Equivocal |
| 37 | 75+ | <20 | 5-7 | Localized | Mod/high | Equivocal | Inappropriate | Appropriate | Equivocal | Equivocal |
|  |  |  |  |  |  |  |  |  |  |  |
| 49 | 75+ | 20-99 | 5-7 | Localized | Low | Equivocal | Inappropriate | Appropriate | Appropriate | Inappropriate |
| 40 | 75+ | 20-99 | 5-7 | Localized | Mod/high | Inappropriate | Inappropriate | Equivocal | Appropriate | Inappropriate |
